# The influence of socio-cognitive mindfulness, moral sensitivity and dementia communication behaviors on dementia nursing performance of nurses in long-term care hospitals: a cross-sectional study

**DOI:** 10.1186/s12912-024-02013-9

**Published:** 2024-05-24

**Authors:** Hyun Ju Bong, Mikyoung Lee

**Affiliations:** 1 Department of Nursing, Seoyeong University, Gwangju, South Korea; 2 Department of Nursing, Dongshin University, 67 Dongshindae-gil, Naju-si, Jeollanam- do, 58245 South Korea

**Keywords:** Dementia nursing performance, Socio-cognitive mindfulness, Moral sensitivity, Dementia communication behaviors

## Abstract

**Background:**

By incorporating socio-cognitive mindfulness which has been under-examined in the nursing field, this study investigated the relationships between socio-cognitive mindfulness, moral sensitivity, dementia communication behaviors, and dementia nursing performance of nurses in long-term care hospitals. This study also examined the factors influencing nurses’ dementia nursing performance.

**Methods:**

A cross-sectional study was conducted with 209 nurses from long-term care hospitals in Korea. Data were collected between August 1 and August 31, 2022. Participants completed the questionnaire assessing their socio-cognitive mindfulness, moral sensitivity, dementia communication behaviors, and dementia nursing performance. Pearson’s correlation and hierarchical multiple regression analysis were performed for data analysis.

**Results:**

Dementia nursing performance of the nurses in long-term care hospitals was positively related to their socio-cognitive mindfulness, moral sensitivity, and dementia communication behaviors. Furthermore, nurses’ dementia communication behaviors, moral sensitivity, and total clinical career, in that order, were found to be the factors influencing their dementia nursing performance.

**Conclusion:**

The findings indicate that the higher socio-cognitive mindfulness, moral sensitivity, and dementia communication behaviors, the higher dementia nursing performance, and that higher dementia nursing performance is associated with better dementia communication behaviors, greater moral sensitivity, and more extensive clinical experiences. This study provides a foundation for developing effective interventions to enhance dementia nursing performance in the future. To improve dementia nursing performance of nurses in long-term care hospitals, it is necessary to improve dementia communication behaviors and moral sensitivity, and prepare multilateral countermeasures to maintain nurses’ clinical careers.

## Introduction

Along with the aging population, the number of dementia patients has also increased rapidly in Korea. The number of dementia patients over 65 years old rose from 259,000 in 2010 to 935,000 in 2022 [1]. Specifically, while the elderly population has increased by 1.6 times, the number of dementia patients has increased by 3.6 times. Due to the rapid increase in the number of dementia patients and the increase in average life expectancy, numerous people with dementia live for a longer period of time compared to the past. Accordingly, the role and expertise of personnel who care for them is becoming more important.

Dementia patients lose their ability to carry out daily life on their own over time, increasing the burden on their family; thus, the care of dementia patients tends to move from home to long-term care hospitals [2]. In addition, dementia patients account for the largest proportion (44.3%) of all patients in long-term care hospitals [3]. Accordingly, there is a mounting demand for nurses in long-term care hospitals to perform professional and skilled nursing practices that can appropriately cope with dementia patients’ physical symptoms and behavioral and psychological problems.

Dementia nursing performance refers to the ability of nurses equipped with professional knowledge and expert nursing skills to quickly and accurately assess dementia patients [4, 5], applying evidence-based nursing interventions and holistic and person-centered approaches [6]. Quality professional dementia nursing performance optimizes patients’ general health outcomes, in particular improving their physical and cognitive functions; this leads to an increased quality of life and well-being for dementia patients [7]. Therefore, nurses are in an important position to provide professional and high-quality nursing performance that can delay the progression of dementia while maintaining cognitive functions and residual abilities in dementia patients [8]. Dementia patients are not good at expressing themselves, and if their feelings or intentions are ignored or misinterpreted, their behavioral symptoms might increase, which could increase caregiver burden and cause exhaustion and depression [9, 10]. These negative emotions can lead to passive attitudes towards nursing dementia patients [11] and ultimately deteriorate the quality of dementia nursing performance [12, 13]. Therefore, it is necessary for nurses to treat dementia patients with positive emotions and attitudes by effectively regulating their own emotions [14].

One effective strategy for emotion regulation is mindfulness, which focuses on current experience and accepts the situation as it is [15]. Mindfulness is classified into meditative mindfulness [15], which is widely researched in nursing [16–20] and socio-cognitive mindfulness [21], which has received little attention in the nursing field. While meditative mindfulness focuses on the present moment without judgment through meditation, socio-cognitive mindfulness emphasizes flexible interactions with the surroundings by enhancing an individual’s openness to external stimuli [22, 23]. By focusing attention on the current situation through various perspectives [24, 25], socio-cognitive mindfulness can reduce negative emotions, such as stress and emotional exhaustion, while enhancing positive emotions, such as the sense of accomplishment and job satisfaction [26, 27]. In addition, interventions based on socio-cognitive mindfulness can be employed in the short term without meditation training [28]. Given that socio-cognitive mindfulness was useful for improving both cognitive and affective empathy [29], we believe that socio-cognitive mindfulness is highly applicable to busy nursing practice settings. Therefore, we applied socio-cognitive mindfulness in this study. The expectation was that socio-cognitive mindfulness would help nurses possess flexible and open attitudes towards nursing dementia patients, thereby reducing their psychological burden and negative emotions; this would ultimately promote effective dementia nursing performance [30].

Moral sensitivity is the ability to recognize and discover conflicts by sensitively identifying problematic situations surrounding patients, and it is a prerequisite for nurses to make ethical decisions [31, 32]. High moral sensitivity helps nurses make responsible decisions in ethical situations that require moral judgment and enables ethical nursing practice [33]. Dementia patients may show psycho-behavioral symptoms such as aggression, care resistance, hallucinations, delusions, or swearing [34]. Thus, most facilities and institutions implement physical restraints or psycho-pharmacological therapies for safety and accident prevention [35]. Such situations in which patients’ autonomy is limited cause nurses to face various ethical issues and conflict situations [35]. In these situations, nurses should promote moral sensitivity to resolve conflicts with confident ethical decision-making and conduct optimal nursing performance [36, 37].

Dementia communication behaviors refers to nurses’ ability to discard prejudice against dementia patients, understand patients’ degree of disability and needs, and communicate with patients according to their level [38]. Dementia patients suffer from psychological atrophy and emotional isolation as their ability to express themselves declines due to deterioration in cognitive functions and language abilities [39]. In addition, due to communication disorders, behavioral and psychological problem behaviors, such as wandering, anxiety, and aggression, become more severe [40], causing various problems for themselves and caregivers [41]. Therefore, nurses’ effective communication behaviors that regulate their emotions, respect dementia patients, and empathize with them play a very important role in dealing with dementia patients [38]. Desirable communication behaviors with dementia patients allow nurses to establish a relationship of mutual trust and provide quality nursing, which can ultimately result in improving patients’ health [42, 43].

Previous research on dementia nursing performance has mainly involved dementia knowledge and attitudes [44], behavioral and psychological symptoms [4, 13, 45], and empathy [45]. As related factors of dementia nursing performance, the present study considered moral sensitivity, dementia communication behaviors, and socio-cognitive mindfulness which has not yet been adequately studied in the nursing field. In particular, this study aimed to investigate the relationships between socio-cognitive mindfulness, moral sensitivity, dementia communication behaviors, and dementia nursing performance of nurses in long-term care hospitals. This study also examined the factors influencing nurses’ dementia nursing performance. Given the limited empirical evidence on the relationships between these constructs, specific hypotheses regarding these relationships were not proposed. Rather, the present study primarily adopted an exploratory approach to examine these relationships. This exploratory study would enrich the existing literature by uncovering potential correlations among these variables, establishing a foundation for future research in this area. This research will provide basic data for seeking strategies to perform effective dementia nursing and developing educational programs to improve the quality of nursing for dementia patients.

## Methods

### Research design

This study employed a cross-sectional design to examine the relationships between the main variables as well as factors influencing nurses’ dementia nursing performance in long-term care hospitals. The Strengthening the Reporting of Observational Studies in Epidemiology (STROBE) checklist for cross-sectional studies [46] was used as a reporting guideline.

### Participants and procedure

The participants in this study were 209 nurses from ten long-term care hospitals located in G metropolitan city in South Korea. They had experience in nursing dementia patients, understood the research purpose, and voluntarily agreed to participate in the study. Nurses who had less than 3 months of clinical experience were excluded. The G*power 3.1.9 software program (University of Düsseldorf, Düsseldorf, Germany) was used to calculate the sample size. The minimum sample size was 178 based on an α probability of 0.05, an effect size of 0.15, a power of 0.95, and 11 predictors in multiple regression. Considering a drop rate of 20%, 220 questionnaires were distributed and 218 were collected. Of these, 209 were used for the analysis, excluding nine with insincere responses.

Data were collected between August 1 and August 31, 2022. The first author visited the nursing departments in the ten long-term care hospitals which were conveniently selected in the metropolitan city. She received their approval of study participation after explaining the research purpose and procedure. The questionnaire was distributed to the nurses in these ten hospitals who voluntarily consented to participate in the study. It took approximately 15 min to complete the questionnaire. The participants each turned in their completed questionnaire in a sealed envelope to their nursing department. The first author later received these questionnaires from the departments.

### Ethical consideration

This study was conducted after acquiring approval from the Institutional Review Board at K University in Korea (1041465-202207-HR-001-24). The participants were informed of the research purpose, participation period, procedure, and personal information security. They were also assured that their data would be kept anonymous and confidential and used solely for the research. They had the right to withdraw from the study at any time, if they wished. A small gift was provided to the participants after they completed the questionnaire.

### Measures

To measure participants’ socio-cognitive mindfulness, the Korean validated Langer Mindfulness Scale (LMS) by Kim [25] was used. The LMS, originally developed by Bodner and Langer [47], consists of four dimensions with 21 items. The four dimensions include novelty seeking with six items, novelty producing with six items, flexibility with four items, and engagement with five items. The participants were asked to rate their level of agreement with each item on a 5-point Likert scale ranging from 1 (*strongly disagree*) to 5 (*strongly agree*). The scores ranged from 21 to 105, with higher scores indicating a greater degree of socio-cognitive mindfulness. Cronbach’s alpha for the original scale was 0.85 [48], 0.90 for the Korean LMS [25], and 0.90 in the present study.

Moral sensitivity was measured using the Korean validated Moral Sensitivity Questionnaire (MSQ) by Han et al. [48], which was originally developed by Lützén et al. [49]. This scale consists of five subscales with 27 items: professional responsibility (seven items), patient-centered care (five items), benevolence (five items), moral conflict (five items), and moral meaning (five items). The participants answered the items on a 7-point Likert scale ranging from 1 (*strongly disagree*) to 7 (*strongly agree*). The scores ranged from 27 to 189; the higher the score, the higher the level of moral sensitivity. Cronbach’s alpha for the original MSQ was 0.78 [49], 0.76 for the Korean MSQ [48], and 0.85 in the present study.

To assess dementia communication behaviors, the Communication Behavior Scale of nurses caring for people with Dementia (CBS-D) developed by Lee and Kang [50] was utilized. This scale is composed of four subscales with 18 items: discourse response management (five items), interpersonal control (three items), emotional expression (six items), and interpretability (four items). The items were rated on a 5-point Likert scale ranging from 1 (*never*) to 5 (*always*). The scores ranged from 18 to 90, with higher scores indicating a greater level of dementia communication behaviors. Cronbach’s alpha for the original CBS-D was 0.88 [50] and 0.90 in the present study.

Dementia nursing performance was measured using a scale initially developed by Hwang and Jang [51], which was subsequently modified and content validated for nurses in long-term care hospitals by Park [52]. Park’s [52] adaptation and validation processes ensured the scale’s suitability and effectiveness within the context of long-term care facilities. This scale includes four dimensions: communication (five items), safety management (six items), health promotion (seven items), and daily life function (three items). Each item was answered on a 4-point Likert scale ranging from 1 (*never*) to 4 (*always*). The scores ranged from 21 to 84; the higher the score, the higher the degree of dementia nursing performance. Cronbach’s alpha for the original scale was 0.83 [51], 0.90 for the modified version [52], and 0.92 in the present study.

In addition, participants’ demographic information was collected using a self-report questionnaire that included gender, age, education level, religion, total clinical career, work experience in long-term care hospitals, position, work pattern, and dementia education experience.

### Data analyses

Data were analyzed using the SPSS 26.0 program (IBM Corporation, Armonk, NY, USA). The general characteristics of the participants and the main study variables (i.e., socio-cognitive mindfulness, moral sensitivity, dementia communication behaviors, and dementia nursing performance) were analyzed with frequency, percentage, mean, and standard deviation. The differences in dementia nursing performance according to the participants’ characteristics were analyzed with the *t*-test and one-way analysis of variance. Scheffé’s test was conducted to identify differences between the groups. Correlations between the main variables were analyzed with Pearson’s correlation coefficient. Finally, hierarchical multiple regression analysis was performed to identify the factors influencing dementia nursing performance.

## Results

### Participants’ characteristics and differences in dementia nursing performance

Table 1 displays the general characteristics of the participants and differences in their dementia nursing performance. The participants were 209 nurses in long-term care hospitals, consisting of 96.2% females and 3.8% males. About 70% of them were over 40 years old, and 55.0% had a bachelor’s degree. Regarding the total clinical career, the average years of clinical experiences was 11.2 years (± 8.76), with the highest (35.4%) being in the range of 10–20 years. The average work experience in long-term care hospitals was 5.5 years (± 4.75), with the highest (26.3%) being in the range of 1–3 years. Most participants were staff nurses (77.5%), and 51.2% were working shift-based. In addition, 78.9% had received dementia education. There was a significant difference in dementia nursing performance in terms of age (F = 6.156, *p <* .001), total clinical career (F = 4.295, *p* = .007), and dementia education experience (*t* = 3.158, *p* = .002).

**Table 1:** Differences in dementia nursing performance according to general characteristics (*n* = 209)

Characteristics	Categories	*n* (%) or M ± SD	Dementia nursing performance		
			M ± SD	*t*/F	*p* (Scheffé)
Gender	Male	8 (3.8)	4.62 ± 0.71	0.679	0.498
	Female	201 (96.2)	4.76 ± 0.55		
Age (years)	< 30^a^	33 (15.8)	2.93 ± 0.28	6.156	< 0.001 (a < c, d)
	30–39^b^	33 (15.8)	3.03 ± 0.34		
	40–49^c^	63 (30.1)	3.17 ± 0.33		
	≥ 50^d^	80 (38.3)	3.20 ± 0.35		
Education level	Associate degree	78 (37.3)	4.71 ± 0.55	1.491	0.228
	Bachelor’s degree	115 (55.0)	4.74 ± 0.54		
	Master’s degree	16 (7.7)	5.07 ± 0.71		
Religion	Christian	60 (28.7)	3.08 ± 0.30	1.115	0.379
	Buddhist	17 (8.1)	3.15 ± 0.36		
	Catholic	24 (11.5)	3.29 ± 0.44		
	No religion	104 (49.8)	3.11 ± 0.33		
	Others	4 (1.9)	3.01 ± 0.41		
Total clinical career (years)		11.2 ± 8.76			
	< 3^a^	37 (17.7)	3.04 ± 0.33	4.295	0.007
	3–< 10^b^	60 (28.7)	3.05 ± 0.30		(a, b < d)
	10–< 20^c^	74 (35.4)	3.12 ± 0.33		
	≥ 20^d^	38 (18.2)	3.30 ± 0.39		
Career in long-term care hospitals (years)		5.5 ± 4.75			
	< 1	25 (12.0)	3.06 ± 0.36	1.344	0.255
	1–< 3	55 (26.3)	3.12 ± 0.33		
	3–< 6	47 (22.5)	3.08 ± 0.35		
	6–< 10	39 (18.6)	3.09 ± 0.32		
	≥ 10	43 (20.6)	3.22 ± 0.36		
Position	Staff nurse	162 (77.5)	3.11 ± 0.35	2.479	0.086
	Charge nurse	6 (2.9)	2.92 ± 0.22		
	Head nurse	41 (19.6)	3.21 ± 0.32		
Work pattern	Shift work	107 (51.2)	3.11 ± 0.36	0.048	0.953
	Day fixed	69 (33.0)	3.13 ± 0.31		
	Night fixed	33 (15.8)	3.12 ± 0.36		
Dementia education experience	Yes	165 (78.9)	3.16 ± 0.34	3.158	0.002
	No	44 (21.1)	2.98 ± 0.32		

*Note* M = mean; SD = standard deviation

### Mean levels of variables

The means and standard deviations are presented in Table 2. The mean levels of all variables were above the midpoints of their respective scales. The mean levels of socio-cognitive mindfulness, moral sensitivity, dementia communication behaviors, and dementia nursing performance were 3.53 (± 0.50), 4.91 (± 0.53), 3.86 (± 0.45). and 3.12 (± 0.34), respectively.

### Relationships between socio-cognitive mindfulness, moral sensitivity, dementia communication behaviors, and dementia nursing performance

Correlations between the study variables are shown in Table 2. The participants’ dementia nursing performance was positively related to socio-cognitive mindfulness (*r* = .498, *p <* .001), moral sensitivity (*r* = .505, *p <* .001), and dementia communication behaviors (*r* = .714, *p <* .001). Socio-cognitive mindfulness was positively related to moral sensitivity (*r* = .424, *p <* .001) and dementia communication behaviors (*r* = .667, *p <* .001). Additionally, moral sensitivity was positively related to dementia communication behaviors (*r* = .551, *p <* .001).

**Table 2:** Correlations, means, and standard deviations for the variables (*n* = 209)

Variables	Socio-cognitive mindfulness		Moral sensitivity		Dementia communication behaviors		Dementia nursing performance
Socio-cognitive mindfulness	1						
Moral sensitivity	0.424***		1				
Dementia communication behaviors	0.667***		0.551***		1		
Dementia nursing performance	0.498***		0.505***		0.714***		1
M ± SD	3.53 ± 0.50		4.91 ± 0.53		3.86 ± 0.45		3.12 ± 0.34
Possible range	1–5		1–7		1–5		1–4

*Note* M = mean; SD = standard deviation. *** *p* < .001

### Factors influencing dementia nursing performance

Hierarchical multiple regression analysis was performed to identify the factors influencing dementia nursing performance. Table 3 presents the results of that regression analysis. Prior to hierarchical regression analysis, the basic assumptions of autocorrelation and multicollinearity between the independent variables were checked. The Durbin-Watson coefficient was 1.82, indicating the absence of autocorrelation. The tolerance limit ranged from 0.45 to 0.95, and the variance inflation factor ranged from 1.05 to 2.23. The correlations between the independent variables were less than 0.80. Therefore, multicollinearity was not an issue in the data [53].

Hierarchical multiple regression analysis was conducted in four steps. In Model 1, age, total clinical career, and dementia education experience, which showed a significant difference in dementia nursing performance among the general characteristics, were entered as dummy variables. All factors of age (β = –0.193, *p* = .006), total clinical career (β = 0.172, *p* = .012), and dementia education experience (β = 0.154, *p* = .022) influenced dementia nursing performance, accounting for 11.6% of the variance (F = 10.096, *p <* .001).

In Model 2, socio-cognitive mindfulness was added to Model 1, controlling for age, total clinical career, and dementia education experience. The factors influencing dementia nursing performance were total clinical career (β = 0.152, *p* = .014) and socio-cognitive mindfulness (β = 0.435, *p <* .001). The variance explained by Model 2 was 28.5% of dementia nursing performance (F = 21.680, *p <* .001).

In Model 3, moral sensitivity was added to Model 2, controlling for age, total clinical career, dementia education experience, and socio-cognitive mindfulness. Total clinical career (β = 0.147, *p* = .011), socio-cognitive mindfulness (β = 0.315, *p <* .001), and moral sensitivity (β = 0.333, *p <* .001) significantly influenced dementia nursing performance. This model showed 37.0% of the variance (F = 25.450, *p <* .001).

In the final Model 4, the factor of dementia communication behaviors was added to Model 3. The result showed that total clinical career (β = 0.124, *p* = .014), moral sensitivity (β = 0.154, *p =* .009), and dementia communication behaviors (β = 0.593, *p <* .001) positively influenced dementia nursing performance. The variance explained by Model 4 was 52.9% of dementia nursing performance (F = 39.970, *p <* .001).

**Table 3:** Hierarchical multiple regression analysis on dementia nursing performance (*n* = 209)

	Model 1				Model 2				Model 3				Model 4		
	β	*t*	*p*		β	*t*	*p*		β	*t*	*p*		β	*t*	*p*
(Constant)		43.344	< 0.001			12.781	< 0.001			6.436	< 0.001			4.969	< 0.001
Age (< 40)	–0.193	–2.783	0.006		–0.084	–1.308	0.192		–0.027	–0.435	0.664		–0.001	–0.028	0.978
Total clinical career (≥ 20)	0.172	2.521	0.012		0.152	2.471	0.014		0.147	2.551	0.011		0.124	2.485	0.014
Dementia education experience (Yes)	0.154	2.306	0.022		0.099	1.629	0.105		0.078	1.364	0.174		0.007	0.149	0.881
Socio-cognitive mindfulness					0.435	7.021	< 0.001		0.315	5.057	< 0.001		0.019	0.288	0.773
Moral sensitivity									0.333	5.361	< 0.001		0.154	2.656	0.009
Dementia communication behaviors													0.593	8.341	< 0.001
*R* ^*2*^	0.129				0.298				0.385				0.543		
*Adj. R* ^*2*^	0.116				0.285				0.370				0.529		
F (*p*)	10.096 (< 0.001)				21.680 (< 0.001)				25.450 (< 0.001)				39.970 (< 0.001)		

*Note* Dummy variables: Age (≥ 40), total clinical career (< 20), dementia education experience (No)

## Discussion

The present study examined the relationships between socio-cognitive mindfulness, moral sensitivity, dementia communication behaviors, and dementia nursing performance among nurses in long-term care hospitals. In particular, this study investigated the factors influencing nurses’ dementia nursing performance. Due to few prior studies utilizing the same variables, the investigation of these relationships in the present study was more likely exploratory in nature. Nevertheless, it remained feasible to discuss the results by referring to studies that have explored similar variables. This study found that all variables of socio-cognitive mindfulness, moral sensitivity, dementia communication behaviors, and dementia nursing performance were positively related to each other. Furthermore, nurses’ dementia communication behaviors, moral sensitivity, and total clinical career were found to be the factors influencing their dementia nursing performance.

First of all, nurses’ dementia nursing performance in long-term care hospitals was positively related to their socio-cognitive mindfulness, moral sensitivity, and dementia communication behaviors. This finding indicates that the higher the level of socio-cognitive mindfulness, moral sensitivity, and dementia communication behaviors, the higher the level of dementia nursing performance. While no studies have examined the same variables as those examined in this study, it is possible to discuss our finding in relation to studies that have investigated similar variables. For example, nurses’ socio-cognitive mindfulness has been found to promote effective emotional regulation and positively correlates with empathy [54]. Socio-cognitive mindfulness might have reduced nurses’ psychological burden and stress resulting from dementia patients’ challenging behaviors by helping to effectively regulate emotions in our participants. This might have positively influenced their dementia nursing performance, as reflected in the present study. In addition, given that empathy towards patients is essential in dementia nursing performance [52], the finding that socio-cognitive mindfulness can enhance empathy [54] highlights its relevance for our study.

Earlier research suggests that moral sensitivity is positively associated with elderly nursing practice, elderly nursing attitudes [55, 56], and person-centered care [57]. These results support the finding of the present study regarding the positive correlation between moral sensitivity and dementia nursing performance among nurses in long-term care hospitals. Since most dementia patients are elderly, dementia nursing should be performed within the framework of elderly nursing. In addition, caregivers may tend to regard dementia patients only as service objects and objectify them, regarding dementia patients as not knowing anything because of their cognitive impairment [58]. Thus, nurses should provide dementia nursing with person-centered care and attitudes; to this end, nurses are required to possess moral sensitivity.

On the one hand, communication skills were positively related to elderly nursing practice [59], caring behaviors [60], and person-centered nursing [61]. In particular, dementia communication behaviors showed a positive correlation with nurses’ empathy [50] and empathy satisfaction [62]. These findings suggest that nurses’ communication skills are important for providing person-centered nursing to elderly patients and that when nurses have high empathy skills, they can communicate better with dementia patients. This will improve nurses’ empathy satisfaction and ultimately lead to better-quality dementia nursing performance.

According to the four-step hierarchical multiple regression analysis, dementia communication behaviors, moral sensitivity, and total clinical career positively influenced dementia nursing performance, in that order. This suggests that a higher level of dementia nursing performance is associated with better dementia communication behaviors, greater moral sensitivity, and more extensive clinical experience.

The finding that dementia communication behaviors was the strongest predictor of dementia nursing performance could be explained by previous studies, revealing that communication skills positively influenced elderly nursing practice [59], person-centered nursing performance [61], and caring behaviors [60]. Although these studies did not examine the same variables as this study, elderly nursing practice, person-centered nursing performance, and caring behaviors [60] include considerable aspects needed for dementia nursing performance, and they are influenced by nurses’ communication skills. Moreover, a previous study found that dementia communication behaviors had a positive effect on nurses’ empathy satisfaction [62]; based on this finding, we could presume that successful communication with dementia patients can promote nurses’ empathy satisfaction, which can potentially enhance dementia nursing performance.

The second factor that influenced dementia nursing performance was moral sensitivity. This result is in line with recent studies, showing that moral sensitivity was positively associated with elderly nursing practice and elderly nursing attitudes [55, 56]. In fact, the questionnaire items to measure dementia nursing performance, elderly nursing practice, and elderly nursing attitudes are similar to some degree. When caring for elderly patients, especially dementia patients with physical and mental weakness, nurses should perform nursing practice with a sense of a morality [55]. As such, it is important for nurses to recognize that moral sensitivity must precede moral dementia nursing performance.

The third factor influencing dementia nursing performance was the total clinical career of nurses, and those over 20 years of experience showed higher dementia nursing performance. This result can be supported by previous findings that the lack of experience in caring for dementia patients increased the burden of dementia behavioral and psychological symptoms [9, 10], and that the greater the amount of clinical experience, the higher the efficiency and proficiency in performing nursing tasks [63]. When providing dementia nursing performance, nurses need to quickly and accurately understand the problems of dementia patients, and they are required to possess professional knowledge and skilled techniques; these can be acquired through a variety of clinical case experiences [8]. As nurses gain more clinical experience, they accumulate lessons and reflections from trial-and-error experiences and successes they experienced during nursing performance [45], resulting in better dementia nursing performance for the nurses in this study as well.

Meanwhile, in Model 2 and Model 3 of the hierarchical regression analysis, socio-cognitive mindfulness had a positive effect on dementia nursing performance, and the variance explained by these two models was 28.5% and 37.0%, respectively. As such, it is difficult to rule out a positive relationship between socio-cognitive mindfulness and dementia nursing performance, although socio-cognitive mindfulness was not an influencing factor in Model 4. Nurses experience negative emotions such as burden, stress, burnout, and depression due to the psycho-behavioral symptoms of dementia patients, which negatively affect dementia nursing performance [4, 13, 30]. In unfavorable situations, socio-cognitive mindfulness, which might lead to effective emotional regulation and psychological well-being through simple cognitive interventions (e.g., recognizing and writing down one’s emotions or feelings for about one minute every day) [22], would help nurses perform high-quality nursing by managing their emotions and enhancing their empathy [54]. Therefore, it is worthwhile to apply socio-cognitive mindfulness to improve dementia nursing performance.

Regarding the insignificant effect of socio-cognitive mindfulness on dementia nursing performance in Model 4, it is difficult to provide a thorough discussion here due to little research on socio-cognitive mindfulness in the nursing literature. One possible reason could be that the significant influence of socio-cognitive mindfulness on dementia nursing performance might have been offset, which could have been a statistical artifact due to the significant correlations between the variables. Future studies might investigate this aspect in more detail.

Furthermore, the present study confirmed that dementia education experience did not influence dementia nursing performance. Previous studies have reported mixed results regarding the relationship between dementia education experience and dementia nursing performance. Our result was consistent with Baek’s [13] finding but inconsistent with other studies [4, 44, 52]. Given that nurses with dementia education experience showed higher dementia nursing performance than those without dementia education experience in our study, and that nurses’ attitudes towards dementia patients and nursing performance both improved after receiving dementia education [64], the importance of dementia education is unquestionable. Nonetheless, the dementia education curriculum in Korea is problematic because courses take place sporadically, and the contents are not systematic, compared to the systematic operation of the dementia education curriculum in the UK or Japan [65]. It is necessary to develop and complement dementia education programs that can be applied directly in practice. For example, integrated dementia education, such as case-based education, role play, and case discussion, will be more helpful than limited theory-oriented education [66].

There are some limitations to be considered when interpreting our findings. First, owing to the nature of the cross-sectional design, our findings on the relationships between the variables should be cautiously interpreted. To obtain a more comprehensive understanding, future research could incorporate individual in-depth interviews or narratives as well as a longitudinal study. Second, the use of a self-reported questionnaire in our study might have introduced bias, as participants, particularly nurses, might have provided socially desirable answers related to mindfulness, moral sensitivity, dementia communication behaviors, and dementia nursing performance. Future research should incorporate complementary methods, such as observational assessments or interviews, to offer a more comprehensive understanding of dementia care in long-term care hospitals. Third, the use of the dementia nursing performance scale, developed and validated within the Korean context, may have constraints regarding its generalizability beyond this population. Although the scale has exhibited reliability within Korean studies, its applicability in international contexts may be questioned due to the lack of validation across diverse cultural backgrounds. Future research could focus on conducting validation studies in various international settings, adapting the scale to different cultural contexts and assessing its psychometric properties to ensure its validity and reliability across diverse populations. Fourth, this study was conducted with nurses in long-term care hospitals in one city in Korea using convenience sampling. Thus, the results may not be generalizable to the broader population of nurses. Repetitive studies with diverse samples from different locations are necessary to gain a better understanding of the relationships between the variables. Finally, there may be other potential factors influencing dementia nursing performance that were not considered in our study. For example, nurses’ stress, due to patients’ severity of dementia or a low ratio of staff compared to the number of patients, could adversely affect dementia nursing performance. Therefore, further investigation is also required in this area.

## Conclusions

By incorporating socio-cognitive mindfulness which has been under-examined in the nursing field, this study explored how socio-cognitive mindfulness, moral sensitivity, and dementia communication behaviors are associated with dementia nursing performance among nurses in long-term care hospitals. This study also identified the factors affecting nurses’ dementia nursing performance. This is the first study verifying the influence of socio-cognitive mindfulness, moral sensitivity, and dementia communication behaviors in relation to dementia nursing performance. We confirmed significant associations between the variables as well as influencing factors of dementia communication behaviors, moral sensitivity, and total clinical career on nurses’ dementia nursing performance. This finding is meaningful in that it provides basic data to serve as a foundation for developing effective interventions to enhance dementia nursing performance in the future.

Nursing for dementia patients is a protracted process that requires a massive amount of time and effort. For dementia patients to maintain optimal health and live a humane life, it is necessary to improve the level of dementia nursing performance of nurses. Thus, nurses should approach dementia patients with constant efforts and interest in improving their dementia nursing performance. Based on the present study, it is necessary to improve dementia communication behaviors and moral sensitivity to improve dementia nursing performance of nurses in long-term care hospitals. Furthermore, it is crucial to prepare multilateral countermeasures, such as practical educational strategies and long-term working environments to maintain nurses’ clinical careers. To this end, collaborative efforts are necessary not only from nurses and nursing leaders in the nursing field but also from institutional authorities to provide comprehensive support.

## Data Availability

The data presented in this study are available upon request from the corresponding author.

## Abbreviations

LMS: Langer Mindfulness Scale
MSQ: Moral Sensitivity Questionnaire
CBS-D: Communication Behavior Scale of nurses caring for people with Dementia
